# Direct observation of V-trimers in the crystal structure of LiVO_2_

**DOI:** 10.1038/s42004-025-01595-y

**Published:** 2025-07-09

**Authors:** S. Yun, C.-F. Chang, S.-H. Chen, C.-Y. Kuo, L. H. Tjeng, A. C. Komarek

**Affiliations:** 1 Max-Planck-Institute for Chemical Physics of Solids, Dresden, Germany; 2 National Synchrotron Radiation Research Center, Hsinchu, Taiwan, ROC; 3 Department of Electrophysics and Center for Emergent Functional Matter Science, National Yang Ming Chiao Tung University, Hsinchu, Taiwan, ROC

**Keywords:** Structure of solids and liquids, Solid-state chemistry, Electronic materials

## Abstract

Although a trimerized ground state has been predicted for LiVO_2_ for over half a century, the direct determination of its crystal structure in the trimerized phase by X-ray diffraction remains elusive. Depending on the cooling process, two distinct structural modifications have been prepared - LiVO_2_ with streak-like, smeared-out superstructure reflection intensities and Li_0.91_VO_2_ with well-separated superstructure reflection intensities. The discovery of well-separated superstructure reflections in Li_0.91_VO_2_ enabled us to solve its crystal structure. This motivated us to attribute the streak-like superstructure reflection intensities in LiVO_2_ to an overlap of reflections due to a unit cell doubling along the *c*-axis, which contrasts with the previous interpretation of randomly stacked V trimer layers. The availability of the crystal structure data forms a starting point for the quantitative modeling of the trimerization phenomenon in this complex Mott insulator.

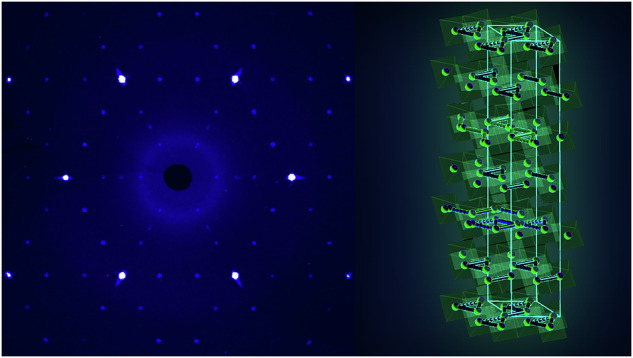

## Abstract (web summary)

LiVO_2_ has been predicted to exhibit a trimerized ground state, but details concerning its crystal structure have remained elusive. Here, the authors investigate the crystal structure of Li_x_VO_2_ (x ≈ 1, 0.91) and find that a V-trimer pattern remains stable even when Li is deficient, with the two compositions crystallizing as distinct polytypes.

## Introduction

The triangular lattice system LiVO_2_ remains an enigmatic material although it has been synthesized already more than 70 years ago^1^. Earlier studies reveal that LiVO_2_ undergoes a structural phase transition at about 463 K^2^. The high-temperature phase adopts a rhombohedral structure with space group $R\bar{3}m$, resembling a derivative of the rock-salt structure, where the (1 1 1) planes are alternatingly occupied by either Li or V ions. Despite numerous structural studies^2–20^, the exact nature of the low-temperature structure remains unsolved. Since the phase transition coincides with a sudden disappearance of the paramagnetic susceptibility signal^2^, it was proposed that nonmagnetic V trimers form in the low-temperature phase^5^. Resistivity measurements have shown that the first-order phase transition is not a conventional metal-to-insulator transition but rather an insulator-to-insulator transition^4,6,8,14^. The “Mottness” of LiVO_2_ complicates interpretations based on a standard Peierls type transition, and a consensus on a quantitative theoretical model has yet to be reached^10,21–26^.

Indications for the existence of non-magnetic trimers in LiVO_2_ have been gathered from various experimental techniques. NMR studies have culminated in the observation of an orbital reconstruction with threefold rotational symmetry in the low-temperature phase^12,15,16,25,27^. X-ray absorption fine structure and pair distribution function (PDF) analyzes have revealed peak splitting associated with the different V–V distances^13,16^, supporting the presence of trimers. X-ray diffraction (XRD) and electron diffraction studies have reported third-integer superstructure reflections (1/3 1/3 *L*) which appear as diffuse streaks along the c*-direction^7–9,11–14,16^. A more recent PDF study using powder XRD suggests that the vanadium trimers are periodically ordered within each *ab*-plane, but that these ordered planes are randomly stacked along the *c*-axis direction^18^. Interestingly, a subsequent study^19^ reported a Li-deficient sample in which powder XRD measurements suggested indications for short-range order in the *c*-direction. However, the crystal structure could not be solved, and no follow-up studies using the more informative single crystal XRD were conducted. Likewise, no further reports used these findings to resolve the crystal structure of stoichiometric LiVO_2_.

Here, we report single-crystal XRD measurements on two Li_1−x_VO_2_ crystals, each representing a different low-temperature modification. One crystal, LiVO_2_, exhibits smeared-out, streak-like superstructure reflection intensities, while the other, Li_0.91_VO_2_, shows well-separated reflections that are broadened along the c*-axis. The presence of well-separated reflections in the latter allowed us to determine its crystal structure. This finding enabled us to reinterpret the streak-like superstructure reflections in our LiVO_2_ sample, attributing them to a unit cell doubling along the *c*-axis, rather than a random stacking of vanadium trimer layers. A structural model for LiVO_2_ was also developed, which provides a reasonable fit to the XRD data despite superstructure reflection overlap and disorder. We note that the correlation length of each reflection in the *c*-direction is similar for both structural modifications, and that their structural relationship suggests that Li_1−x_VO_2_ is a polytypic compound.

## Results and discussion

**Fig. 1:**
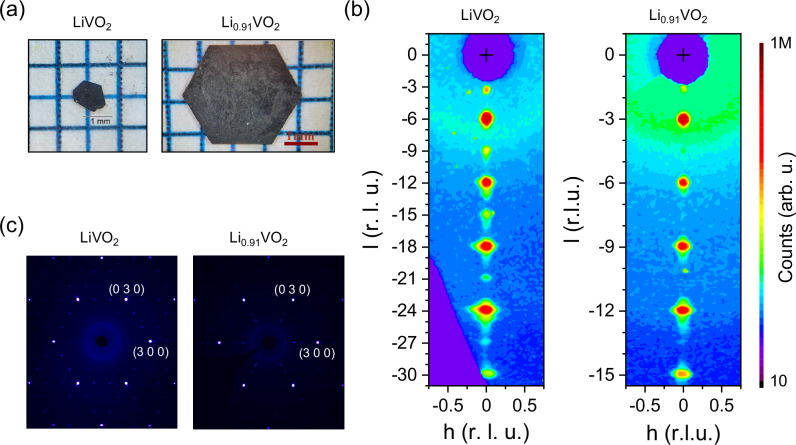
Crystals & characterization. **a** As-grown Li_1−x_VO_2_ single crystals: LiVO_2_ (left) and Li_0.91_VO_2_ (right). **b** X-ray scattering intensities in the (*h*0*l*) plane of reciprocal space measured by means of single crystal X-ray diffraction on LiVO_2_ (left) and Li_0.91_VO_2_ (right). Here, a unit cell doubling in *c*-direction has been already considered for the panel of LiVO_2_. (Unphysical narrow peaks of one or two detector pixels solely are due to detector noise/stingers/cosmic radiation and have been partly removed). **c** X-ray scattering intensities in the (*hk*0) plane of reciprocal space for LiVO_2_ (left) and Li_0.91_VO_2_ (right).

**Fig. 2:**
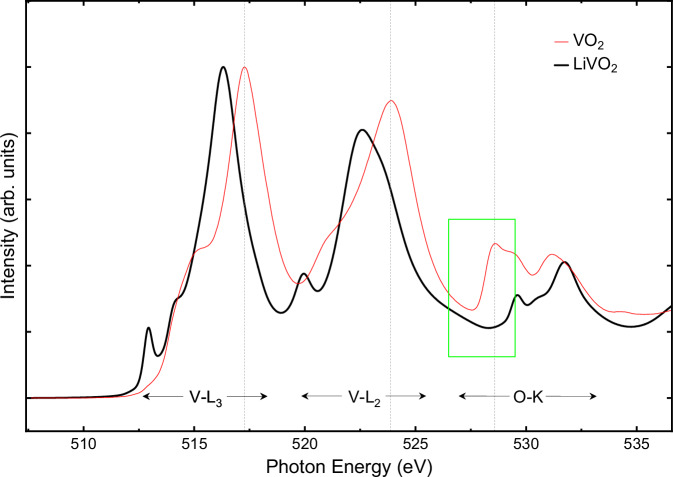
XAS measurements. XAS spectra of LiVO_2_ at the V *L*_2,3_ edges (510–527 eV) and at the O *K* edge (527–537 eV). The black curve shows the spectrum of our full LiVO_2_ compound. The red curve is that of VO_2_. In the inset (green box), we focus on the pre-edge region of the O *K* edge. While VO_2_ (V^4+^) has its main peak at 528.6 eV, LiVO_2_ has there its minimum intensity.

As described in the “Methods” section, we successfully synthesized Li_1−x_VO_2_ single crystals using an optimized flux growth method and tuned the Li content by utilizing different cooling rates during the synthesis. Photos of two single crystals are shown in Fig. 1a. The cooling rate was 10 K/h for LiVO_2_, and 5 K/h for Li_0.91_VO_2_, and the Li content was determined by comparing and calibrating the measured *c*/*a* ratios with literature values for the Li_1−x_VO_2_ system^20^. The Li content of Li_0.91_VO_2_ was also determined by refinements of the Li/V content in single crystal XRD measurements as is discussed below in detail. Representative X-ray scattering intensities are shown in Fig. 1b, c. The stoichiometry of the full LiVO_2_ compound was confirmed by soft X-ray absorption spectroscopy (XAS) measurements at the V L_2,3_ edges (510–527 eV) and at the O *K* edge (527–537 eV), as shown in Fig. 2. The distinct line shapes clearly differentiate the V^3+^ spectrum of LiVO_2_ from the V^4+^ spectrum of VO_2_. Notably, the LiVO_2_ spectrum shows no indications of V^4+^ species.

**Fig. 3:**
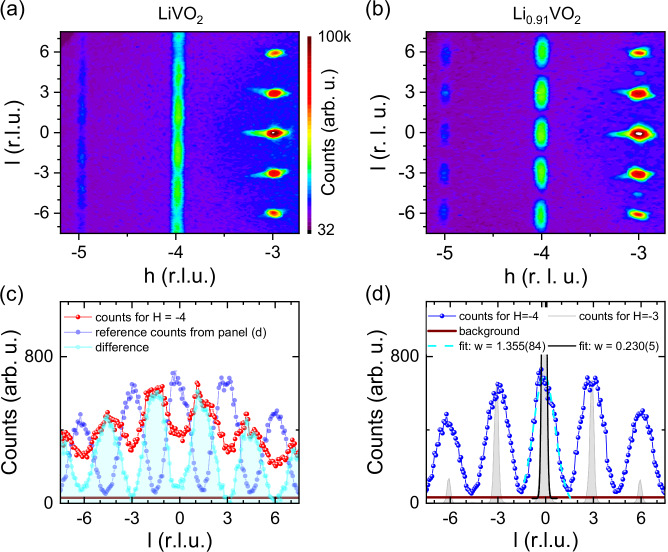
XRD measurements. X-ray scattering intensities in the (*h*0*l*) plane of reciprocal space measured by means of single crystal X-ray diffraction on (**a**, **c**) LiVO_2_ and (**b**, **d**) Li_0.91_VO_2_. The detector background offset amounts to 32 counts; the intensities for both compounds are presented on the same unit cell (without any doubling of **c**); intensity scale is plotted with a logarithmic color scheme. For the single crystal of Li_0.91_VO_2_, the superstructure reflections (at *h* = −4) are well-separated with peak intensities reaching the background level between two reflections. *l*-scans across the superstructure reflections at *h* = −4 are shown in (**c**) for LiVO_2_ (red circles) and in (**d**) for Li_0.91_VO_2_ (blue dots); brown line: detecor background offset. In (**d**), the light gray shaded area indicates a scan across the fundamental Bragg reflections (with intensities divided by 50) and solid lines indicate gaussian fits in order to obtain the peak widths (FWHM). In (**c**), the intensity difference of the intensities of the LiVO_2_ sample and of the intensities of the Li_0.91_VO_2_ sample weighted by 45% is shown. This suggests a doubling of the unit cell in *c*-direction for LiVO_2_ (see text).

Single crystal XRD intensities within the (*h*0*l*) plane of reciprocal space are shown for both samples in Fig. 3a, b. For LiVO_2_ a streak-like intensity distribution in c*-direction is observed, see Fig. 3a, consistent with previous reports in literature^18^. At first glance this pattern suggests a rather high degree of disorder or that the “*vanadium trimers are randomly aligned along the c-axis*”^18^. Such a diffuse diffraction pattern poses a significant challenge in solving the underlying crystal structure.

In contrast, the other sample, Li_0.91_VO_2_, shows well-separated superstructure reflection intensities, as shown in Fig. 3b, which are exceptional for Li_1−x_VO_2_. Note that indications for the possible development of short-range order—depending on the cooling process—were also observed in literature^19^. Therein, a completely disordered Li_1−x_VO_2_ sample and a sample with indications for short-ranged correlations in the stacking were reported^19^. A closer look at the X-ray intensities of our LiVO_2_ sample along the (−40*l*)-direction in reciprocal space—see the curve with the red dots in Fig. 3c—reveals that the intensity remains high for all *l* values, while in Li_0.91_VO_2_ the intensities drop to the background level between the peaks—see the curve with the blue dots in Fig. 3d. This allows us to properly integrate  these well-separated superstructure reflection intensities, enabling us to solve the crystal structure of Li_0.91_VO_2_, which is described below. Compared to the fundamental Bragg reflections, the superstructure reflections are nearly six times broader in the c*-direction, indicating the short-ranged nature of the stacking of vanadium layers in the *c*-direction.

**Fig. 4:**
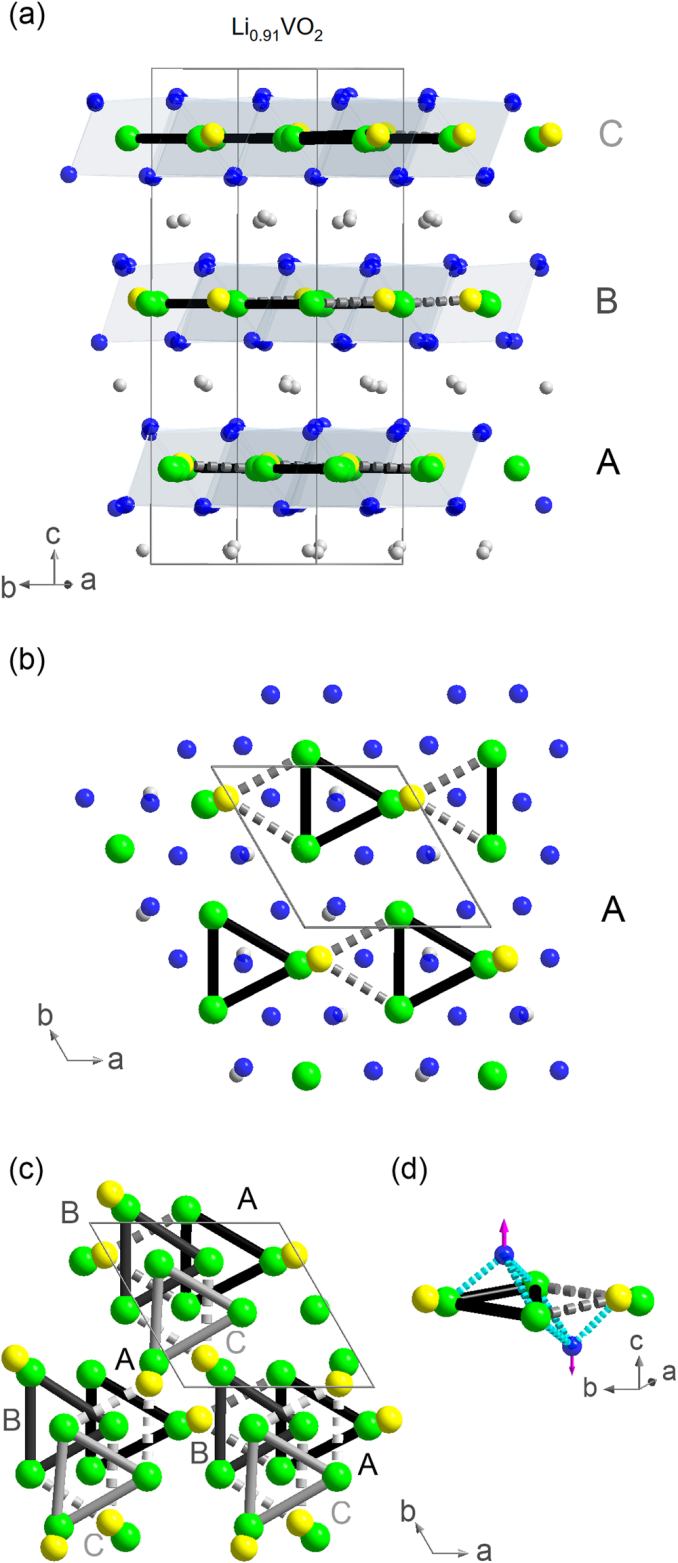
Crystal structure of Li_0.91_VO_2_. The crystal structure model used in the refinement of the single-crystal X-ray diffraction data of Li_0.91_VO_2_. Green/blue/gray spheres denote V-, O- and Li-ions; the yellow spheres indicate V-sites occupied with 24.0(3)% probability (V3' in a split-atom model). The “regular” V-trimers are indicated by thick solid lines and the “flipped” trimers involving the V3' ions are denoted by thick dashed lines. **a** Crystal structure with viewing direction parallel to the vanadium oxide layers A, B, and C. **b** Top view of the single vanadium oxide layer A. **c** Visualization how the trimers in vanadium oxide layers A, B, and C are stacked in *c*-direction. **d** A “regular” trimer together with a “flipped” trimer. The arrows pointing up (down) denote shifts of the oxygen ions that are situated directly over (below) the center of the “regular” (“flipped”) vanadium trimers that appear with a probability of 76.0(3)% (24.0(3)%).

For solving the crystal structure, a total of 14,665 reflections were collected up to 2Θ_*max*_ = 70.8°—see Supplementary Table S1. Apart from the triclinic and trigonal Laue symmetries ($\bar{1}$, $\bar{3}$ and $\bar{3}1m$), all other symmetries can be directly discarded due to extremely high *R*_*int*_-values (>50%) as shown in Supplementary Table S5. (Note that in general, the highest symmetries that appear to be suitable from a comparison of *R*_*int*_-values may not reflect the true crystal symmetries, e.g., due to twinning in the measured crystal that mimics these higher symmetries. The choice of the space group ultimately also depends on the *R*-values and stability of the structure refinements.) Especially, a structural model based on space group *P*31*m* with Laue symmetry $\bar{3}1m$ (and with structural parameters taken from ref. ^18^) would result in *R*-/*R*_*w*_-values of 50.67%/74.3% and a goodness of fit (*GoF)* of 7.37. The best structure solutions yielding the lowest *GoF* and *R*-values were achieved with trigonal space groups with point group $\bar{3}$: i.e., *P*3, $P\bar{3}$ and *P*3_1_ (or *P*3_2_). Among these, the best refinements were achieved with space group *P*3_1_ (or *P*3_2_). Notably, the experimentally observed extinction conditions of systematically absent reflections are also consistent with this space group, as shown in Fig. 1b (right). For reliability, we performed a refinement using only isotropic displacement parameters *U*_*iso*_. Additionally, *U*_*iso*_ was constrained to the same value for each type of element—lithium, vanadium, and oxygen. For light lithium ions, a refinement of *U*_*iso*_ appears to be a natural choice. For oxygen, which is also relatively light and occupies multiple sites within the unit cell, this approach remains reasonable, especially given the complexity of the crystal structure. Since a vanadium site needs to be treated with a split-atom model (see below) and since the crystal exhibits twinning, it is also appropriate to use *U*_*iso*_ for the vanadium ions. Moreover, since all lithium and vanadium ions share a similar octahderal oxygen coordination, applying the same *U*_*iso*_ value for each element type is a reasonable choice. The crystals are twinned with a twin fraction of 49.972(23)% (~racemic mixture of *P*3_1_ and *P*3_2_). This twinning is probably a consequence of stacking faults and the limited correlation length of the stacking of the trimerized layers in the *c*-direction. In addition to this twinning, one of the trimerized vanadium ions, V3, can be found with a probability of 24.0(3)% at a site V3’ effectively flipping or mirroring the trimer to the other side, as shown in Fig. 4a–c. A split-atom model best accounts for this property, which may arise from additional disorder in the stacking of layers. The two different types of trimers are highlighted in Fig. 4a–c with solid lines (“regular” trimers with 76.0(3)% probability) or dashed lines (“flipped” trimers with 24.0(3)% probability). The introduction of the split-atom model for V3 significantly improved the *GoF* and *R*- / *R*_*w*_-values. Otherwise, these values would increase to 2.44 and 8.96%/22.25% for *I* > 3*σ*(*I*) (and 11.09%/25.09% for all reflections). Flipping the trimers alters the stacking of adjacent vanadium layers, which is associated with a completely different kind of stacking fault, unrelated to twinning of *P*3_1_ and *P*3_2_. (But due to the twin law, the trimers are also modeled as being flipped in another direction.) In this case, where the trimers in one layer are “flipped” with a probability of 24.0(3)%, the trimers in two adjacent layers adopt a non-helical arrangement, with a 180° rotation (around *c*) between them. The superstructure reflections are elongated only in the c*-direction and remain sharp in perpendicular directions, indicating that the trimerized patterns are long-range ordered within the vanadium-oxygen planes. The effective lithium deficiency of Li_0.91_VO_2_ is best described with additional vanadium ions at the octahedrally coordinated Li sites which has been also reported in the literature for severely delithiated Li_0.22_VO_2_^28^. Regarding the fact that the refinement only contains three displacement parameters, *U*_*iso*_(Li), *U*_*iso*_(V) and *U*_*iso*_(O), and regarding the disorder in the stacking of layers (indicated by the enhanced detected peak widths in c*-direction), we consider the finally obtained *R*-values and *GoF* acceptable. The structural parameters are listed in Supplementary Table S2. Furthermore, our refinements yield a composition that is consistent with the composition of Li_0.91_VO_2_ derived from its lattice parameters, as determined by the study of lattice parameters and Li content using inductively coupled plasma optical emission spectrometry in literature^20^—the Li:V ratio amounts to 0.932(4) according to our structural refinement. This result is based on the refinement of the occupancy of the comparatively heaviest ions in the system—the V ions—within the Li-deficient layers, and not only on the occupancy refinement of the very light Li ions. The bond valence sums (BVS) for the Li ions are 1.08(1), 1.09(1) and 1.00(1) for Li1, Li2 and Li3, respectively. The BVS for the V ions amount to 2.96(2), 3.02(1) and 3.04(2) for V1, V2 and V3. These values underline the high reliability of our crystal structure refinement.

The V-trimerization is evident from the shortening of the V–V intra-trimer bond lengths: the average intra-trimer V–V distance of 2.59(6) Å is distinctly smaller than the average inter-trimer distance of 2.98(7) Å. Although the stacking of the vanadium trimers is not fully long-range ordered and stacking faults are expected, as indicated by the broadness of the superstructure reflections, there appears to be a preferred stacking order in Li_1−x_VO_2_. It follows an ordering scheme that can be best described by space group *P*3_1_. An illustration of how the presence of trimers in one layer affects the arrangement of trimers in adjacent vanadium layers is provided in Fig. 4d. One oxygen ion is situated above each “regular” trimer and one below each “flipped” trimer. Due to the short V–V distances within each trimer, these oxygen ions are displaced in the out-of-plane direction away from these trimers, as indicated by the arrows in the Fig. 4d. Thus, a potential is created to the adjacent Li oxide layers, and can propagate further along the *c*-direction to subsequent layers. Since no split-atom model was used for these oxygen ions, their differing out-of-plane shifts are a consequence of the probabilities of finding a trimer below/above these ions. In contrast to LiVS_2_^18^, the Li oxide layers in Li_1−x_VO_2_ appear unable to fully transmit these distortions to the next V layer. This limitation could explain the short-ranged nature of the trimer layer stacking. Nevertheless, the stacking is not random and predominantly follows the symmetries of space group *P*3_1_.

**Fig. 5:**
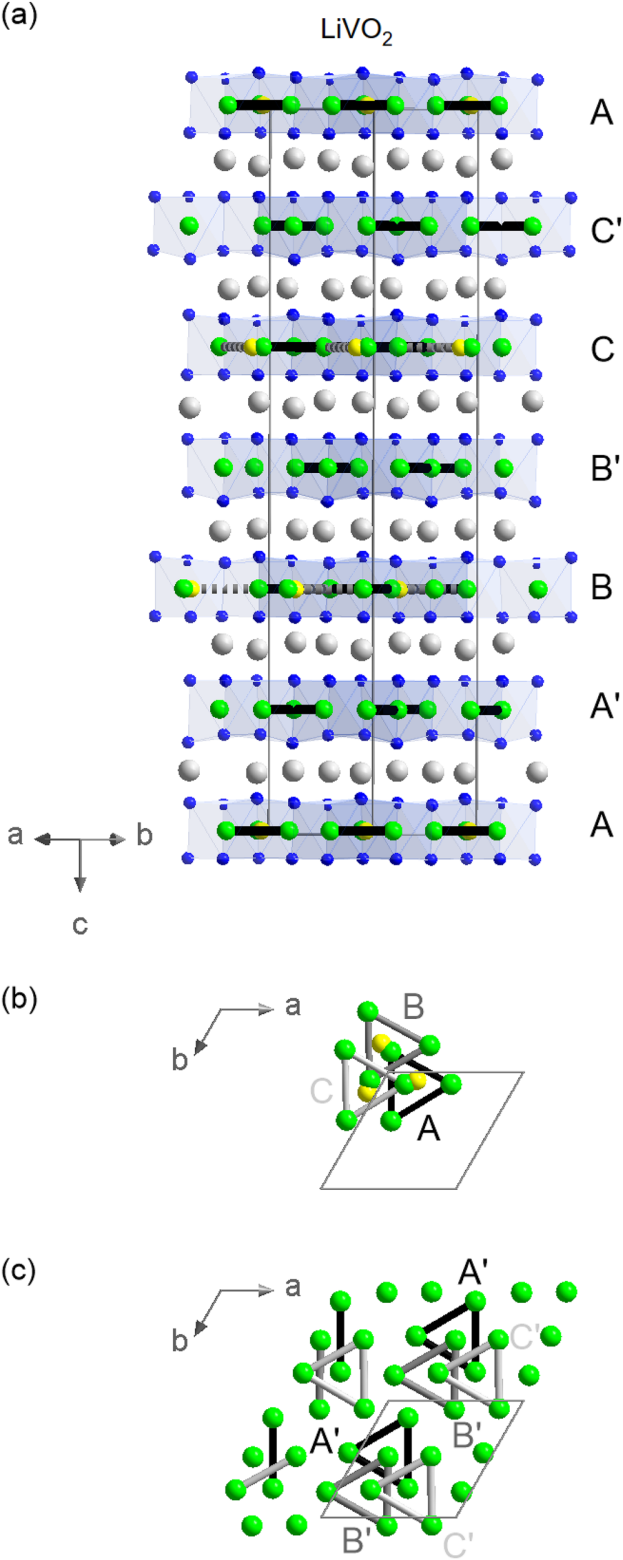
Crystal structure of LiVO_2_. The crystal structure model used in the refinement of the single-crystal X-ray diffraction data of LiVO_2_. Green/blue/gray spheres denote vanadium, oxygen, and lithium ions; the yellow spheres indicate vanadium sites within a split-atom model that are occupied with 28.1(8)% probability. The vanadium trimers involving V3 are indicated by thick solid lines and the alternative trimers involving the V3' ions are denoted by thick dashed lines. **a** Crystal structure with viewing direction parallel to the vanadium oxide layers A, A', B, B', C, and C'. **b** Visualization of how the trimers in vanadium oxide layers A, B & C are stacked in *c*-direction. **c** Visualization of how the trimers in vanadium oxide layers A', B' & C' are stacked in *c*-direction.

After solving the crystal structure of Li_0.91_VO_2_, a crystal structure solution for the LiVO_2_ sample with diffuse superstructure reflection intensities becomes feasible. As shown in Fig. 3c, the superstructure reflections are no longer centered anymore at multiples of three in the (-40*l*)-direction, as in the case of Li_0.91_VO_2_, compare Fig. 3d. Instead, these intensities appear at multiples of 3/2 in the (-40*l*)-direction. A closer inspection of the peak shapes suggests that the reflections in LiVO_2_ are not simply infinitely broad in the c*- direction; rather, the upper parts of these reflections resemble those of Li_0.91_VO_2_. This can be seen when comparing the red and the dark blue data points in Fig. 3c. A simple subtraction of the superstructure reflection intensities of Li_0.91_VO_2_ (weighted by 45%) from the corresponding intensities of LiVO_2_ yields an intensity difference (light blue data) that strongly resembles the superstructure reflection intensities in Li_0.91_VO_2_, including a very similar peak shape and width. The intensity difference shows peaks at *L* values that are multiples of 3/2 with similar peak widths as the superstructure reflections observed for Li_0.91_VO_2_. Our results suggest that the streaks for LiVO_2_ are not caused by a random stacking in the *c*-direction. Instead, the measured intensities for our sample can be interpreted as a superposition of superstructure reflections resulting from a unit cell doubling in the *c*-direction. And indeed, an integration of the XRD intensities in LiVO_2_ with a doubled *c*-lattice constant enabled us to find a structural model for this modification of Li_1−x_VO_2_ that describes the measured XRD intensities reasonably well. This structural model for LiVO_2_ strongly resembles the model found for Li_0.91_VO_2_, see Fig. 5a–c.

For the LiVO_2_ sample, 10458 reflections were measured up to $\sin (\Theta)/\lambda$ = 0.666—see Supplementary Table S3. Similarly to the other sample (Li_0.91_VO_2_), the crystal structure of LiVO_2_ can be described best with the same space group *P*3_1_, twinning, and with similar split-atom model for one of the Vanadium sites. (The experimentally observed extinction conditions are consistent with this space group, as shown in Fig. 1b (left). In the same way as for the other sample, all other symmetries apart from the triclinic and trigonal Laue symmetries $\bar{1}$, $\bar{3}$ and $\bar{3}1m$ could be directly excluded due to their extremely high *R*_*int*_-values > 50%. Moreover, the structure refinement with space group *P*3_1_ was stable, yielding the smallest possible *R*-values and *GoF* values, and without any nonphysical structural parameters.) For the LiVO_2_ sample, the twin fraction amounts to 49.53(14)%, and the split vanadium ion is found with a probability of 71.9(8)% at its regular position, both of which resemble the values determined for the Li_0.91_VO_2_ sample. Refinement and structural parameters for the LiVO_2_ sample can be found in Supplementary Table S4. Considering (i) the complex structure with large unit cell dimensions, (ii) the use of only three isotropic displacement parameters *U*_*iso*_(Li), *U*_*iso*_(V), *U*_*iso*_(O) throughout the entire refinement, (iii) the presence of broad, overlapping superstructure reflections, and (iv) the disorder in the stacking of trimerized layers, the *GoF* and *R*-values attain acceptable values, indicating that the structural model for LiVO_2_ describes the measured X-ray intensities reasonably well. This is further corroborated by the BVS for all the vanadium sites (V1 to V6), which amount to 2.95(4), 2.86(4), 3.06(4), 2.90(3), 2.87(4) and 3.00(4). The vanadium–vanadium distances are listed in Table 1. The intra-trimer V–V distance in Li_1.0_VO_2_ has been directly determined by single crystal XRD, with a value of 2.53(3) Å. In contrast, the distance between non-trimerized neighboring V-ions amounts to 3.00(4) Å. The small scattering of V–V distances among V-ions within trimers and between neighboring V-ions in different trimers provides further evidence for the high reliability of this crystal structure model, as shown in Table 1. With a V–V distance of 2.53(3) Å, the V–V distances within each trimer are even shorter than in vanadium metal, where the shortest V–V distance amounts to 2.6206(5) Å^29^. Such a short bond length would usually favor a picture in which molecular orbitals form within the trimers. However, whether this molecular orbital description is appropriate is an important question, especially for compounds where Mott physics plays a significant role, as discussed above^10,21–26^.

**Table 1:** Vanadium–vanadium distances in LiVO_2_

atoms	distance (Å)	distance (Å)	
	(intra- trimer)	(inter-trimer)	
V1–V2	2.559 (3)	3.036 (2)	2.930 (4)
V1–V3	2.494 (4)	2.964 (3)	3.077 (5)
V2–V3	2.521 (5)	2.995 (4)	3.013 (3)
V4–V5	2.509 (2)	3.045 (3)	2.977 (2)
V4–V6	2.565 (3)	2.973 (5)	2.985 (3)
V5–V6	2.507 (4)	3.031 (3)	2.992 (4)
Average	2.526 (28)	3.00 (4)	

The intra-trimer and inter-trimer V–V distances in LiVO_2_ are listed. Finally, also the statistical average of the intra-trimer and inter-trimer V–V distances together with the statistical error is listed.

**Fig. 6:**
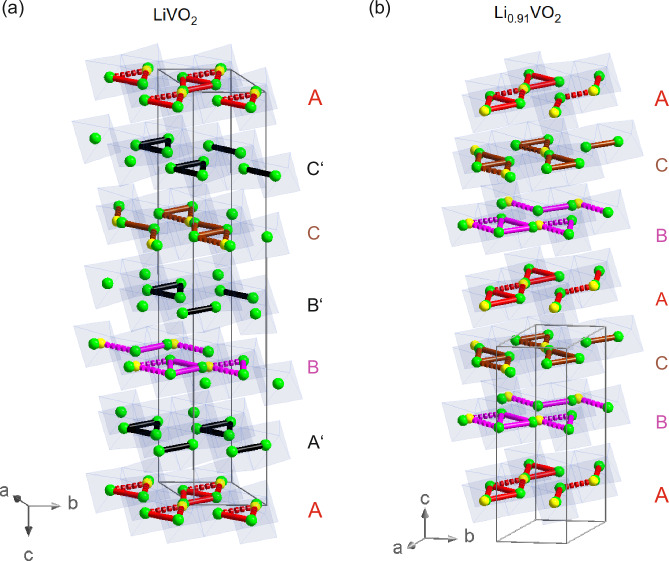
Side-by-side comparison. Comparison of the crystal structures of Li_1−x_VO_2_ for (**a**) LiVO_2_ and (**b**) Li_0.91_VO_2_. Green/yellow spheres denote vanadium ions. The “regular” and “flipped” trimers are represented by thick lines and dashed lines, respectively. Blue octahedra represent the VO_6_ octahedra.

A comparison of the crystal structures of LiVO_2_ and Li_0.91_VO_2_ reveals that the former shares a similar stacking of vanadium layers with the latter: the same structural elements are found in the same stacking order in every second vanadium layer A, B, and C of the LiVO_2_ sample, with the difference that additional vanadium oxide layers A’, B’, and C’ are introduced between the layers A, B, and C, i.e., layer A’ after layer A, etc.—see Fig. 6a, b. This difference in the stacking is responsible for the doubling of the *c*-axis in LiVO_2_. Interestingly, the additionally introduced vanadium layers A’, B’, and C’ do not exhibit any indication of split V-sites, suggesting that there is no related disorder due to a flipping of the trimers within a layer for these vanadium oxide layers A’, B’, and C’ in this structural modification.

## Conclusion

In conclusion, two different Li_1−x_VO_2_ single crystals were synthesized with an optimized flux growth method. One crystal, LiVO_2_, exhibits well-known diffuse, streak-like superstructure reflection intensities, while the other crystal, Li_0.91_VO_2_, has well-separated superstructure reflection intensities. The crystal structure of the latter crystal, Li_0.91_VO_2_, was determined in the trimerized phase. Rather than resulting from a random stacking of the vanadium oxide layers, the streak-like appearance of the superstructure reflection intensities in the other modification of LiVO_2_ can be attributed to a doubling of the *c* lattice constant, which leads to a partial overlap of the doubled amount of superstructure reflections. The peak widths of the superstructure reflections are similar in both modifications, indicating a limited correlation length in the *c*-direction. A structural model for LiVO_2_ was also developed, which provides a reasonable fit to the measured XRD data, despite the overlap of superstructure reflection intensities and disorder. The limited correlation length in the *c*-direction for both structural modifications is consistent with a split-atom model and twinning. Trimer formation was observed at the atomic level in both types of Li_1−x_VO_2_ compounds, including the V–V distances within and between the trimers. Within a trimer of LiVO_2_, the V–V distances of 2.53(3) Å are smaller than those in vanadium metal. Our results also show that the trimer pattern in Li_1−x_VO_2_ remains stable when Li is deficient. The intra-trimer V–V distance is 2.59(6) for Li_0.91_VO_2_. Future theoretical studies will benefit from our crystal structure data on Li_1−x_VO_2_ single crystals, which will help to quantify the extent to which a molecular orbital description of the V-trimers is adequate in view of the Mott physics present in this compound.

## Methods

### Chemical synthesis and characterization

Single crystals of Li_1−x_VO_2_ were grown by a flux method. The starting materials Li_2_CO_3_ (99.995%, Alfa Aesar) and V_2_O_3_ (99.99%, Thermo Fisher) were mixed in stoichiometric quantities (plus a 3% excess of Li_2_CO_3_) and reacted (by solid-state reaction) in an alumina crucible under a flow of reforming gas (5% H_2_ 95% Ar) at 750 °C for 48 h. As a flux for the single crystal growth a mixture of Li_2_CO_3_ and LiBO_2_ was chosen with a mass ratio of LiBO_2_, Li_2_CO_3_ and LiVO_2_ of 10:3:1. The entire mixture (flux and precursor) was heated at 1000 °C for 12 h. Then, it was cooled down to 700 °C with a cooling rate of either 5 K/h (Li_0.91_VO_2_) or 10 K/h (LiVO_2_), and afterwards to 30 °C with a cooling rate of 10 K/h. The whole growth process was conducted in a reforming gas atmosphere. Then, flux and single crystals were separated by dissolving the flux with distilled water at room temperature. For a determination of the Li content *x* we made use of the *c*/*a*-ratio (in notation of the high-temperature $R\bar{3}m$ structure). It has been reported that *a* is smallest and *c* is largest for the (near) stoichiometric compound with *x*~1^20^. The room-temperature lattice parameters of Li_0.91_VO_2_ & LiVO_2_ are listed in Supplementary Tables S1; S3. After transformation to the high temperature ($R\bar{3}m$) unit cell the *c*/*a* ratio of these lattice parameters amounts to 5.2106(5) for Li_0.91_VO_2_ and 5.2405(4) for LiVO_2_. From the former value, we derived the composition of Li_0.91_VO_2_. Note that our XRD results are indicative for a somewhat higher Li content with a composition of Li_0.932(4)_VO_2_. The value for the other sample is somewhat higher than the highest values reported for essentially stoichiometric samples in literature^20^. Since the largest *c*/*a* ratio can be expected for stoichiometric samples, this result would indicate a composition close to LiVO_2_ for that sample. Further complementary XAS measurements of this LiVO_2_ sample confirm its stoichiometry (see main text).

### Single crystal X-ray diffraction (XRD)

Single crystal XRD measurements have been performed on a *Bruker D8 VENTURE* diffractometer (Mo *K*_*α*_ radiation) with a bent graphite monochromator for about three times intensity enhancement and equipped with a *Photon III* CMOS area detector. Single crystal XRD measurements reveal two different types of Li_1−x_VO_2_ systems with different stacking of vanadium oxide layers, which is reflected in the fact that the *c*-lattice constant of the LiVO_2_ sample is doubled compared to the Li_0.91_VO_2_ sample. The reflection conditions of both measured Li_1−x_VO_2_ polytypes are in agreement with space group *P*3_1_, i.e., for (0 0 *l*) only reflections for *l* = 3*n* ($n\in {\mathbb{Z}}$) are observed in both cases—see Fig. 1b. The CIF-files of the structural models that we developed for both polytypes of Li_1−x_VO_2_ can be found in the Supplementary Data 1 (Li_0.91_VO_2_) and Supplementary Data 2 (LiVO_2_). The numerical data of the graphs/plots can be found in the Supplementary Data 3.

### X-ray absorption spectroscopy (XAS)

Soft XAS measurements were conducted in the total electron yield mode at the TPS45A beamline of the National Synchrotron Radiation Research Center in Taiwan. The XAS measurements were performed at 300 K. A fresh sample surface was prepared by top-post-cleaving in ultra-high vacuum at 300 K. A V_2_O_3_ sample was measured simultaneously in an upstream chamber for energy calibration of the V *L*-edges. The numerical data of Fig. 2 can be found in the Supplementary Data 3.

## Supplementary information

Supplementary Information

Description of Additional Supplementary Files

Supplementary Data 1

Supplementary Data 2

Supplementary Data 3

## Data Availability

The source data for this paper are provided in Supplementary Data 1–3: structural refinement data (Supplementary Data 1 and 2) and numerical data for graphs and plots (Supplementary Data 3).

## Code availability

Computer code is available from http://jana.fzu.cz(*Jana2006* version 04/12/2022).

## References

[1] Rüdorff, W. & Becker, H. Notizen: Über Umsetzungen des Vanadin(III)oxyds und des Vanadin(IV)oxyds mit einigen Metalloxyden. *Z. f.ür. Naturforsch. B* **9**, 613–614 (1954).

[2] Bongers, P. F. Structuur en magnetische eigenschappen van enkele complexe oxyden der overgangselementen. *’S-Gravenhage: Excelsior*, Ph.D dissertation, The University of Leiden, Leiden, The Netherlands (1957).

[3] Rüdorff, W. & Becker, H. Notizen: Die Strukturen von LiVO_2_, NaVO_2_, LiCrO_2_ und NaCrO_2_. *Z. f.ür. Naturforsch. B* **9**, 614–615 (1954).

[4] Reuter, B., Weber, R. & Jaskowsky, J. Über Oxidsysteme mit Übergangsmetallen in verschiedenen Oxydationsstufen und ihr elektrisches Verhalten I. Das System VO—LiVO_2_*Z. Elektrochem.* **66**, 832-838 (1962).

[5] Goodenough, J. B. *Magnetism and The Chemical Bond* (Interscience Publishers, 1963).

[6] Kobayashi, K., Kosuge, K. & Kachi, S. Electric and magnetic properties of Li_*x*_V_2−*x*_O_2_. *Mater. Res. Bull.* **4**, 95–106 (1969).

[7] Hewston, T. A. & Chamberland, B. L. Preparation of LiVO_2_ crystals. *J. Solid State Chem.* **59**, 168–173 (1985).

[8] Hewston, T. A. & Chamberland, B. L. A study of the ternary oxide LiVO_2_ and its anomalous behavior. *J. Solid State Chem.* **65**, 100–110 (1986).

[9] Cardoso, L. P., Cox, D. E., Hewston, T. A. & Chamberland, B. L. Structural studies of Li_0.7_VO_2_ in the temperature range 20–300 ^∘^C. *J. Solid State Chem.* **72**, 234–243 (1988).

[10] Goodenough, J. B., Dutta, G. & Manthiram, A. Lattice instabilities near the critical V-V separation for localized versus itinerant electrons in LiV_1−y_M_y_O_2_ (m=cr or ti) li_1-x_vo_2_. *Phys. Rev. B* **43**, 10170–10178 (1991). 10.1103/physrevb.43.10170 9996734

[11] Imai, K., Koike, M., Sawa, H. & Takei, H. Structural change in Li_1−*x*_VO_2_ (x≈0.2) single crystals. *J. Solid State Chem.* **102**, 277–280 (1993).

[12] Onoda, M. & Inabe, T. Role of structural change in phase transition in LiVO_2_. *J. Phys. Soc. Jpn.* **62**, 2216–2219 (1993).

[13] Imai, K., Sawa, H., Koike, M., Hasegawa, M. & Takei, H. Superstructure analyses on single crystals of Li_0.8_VO_2_. *J. Solid State Chem.* **114**, 184–189 (1995).

[14] Tian, W. et al. Single crystal growth and characterization of nearly stoichiometric LiVO_2_. *Mater. Res. Bull.* **39**, 1319–1328 (2004).

[15] Takao, K. & Onoda, M. Li local configurations for the trimerized state of the geometrically frustrated triangular lattice system Li_1-*x*_VO_2_ with 0 ≤ x ≤ 0.14. *J. Phys.: Condens. Matter* **22**, 116003 (2010). 10.1088/0953-8984/22/11/116003 21389478

[16] Pourpoint, F. et al. New insights into the crystal and electronic structures of Li_1+*x*_V_1−*x*_O_2_ from solid state NMR, pair distribution function analyses, and first principles calculations. *Chem. Mater.* **24**, 2880–2893 (2012).

[17] Gaudet, J. & Dahn, J. Lattice constant anomaly in the Li_1+*x*_V_1−*x*_O_2_ system near *x* = 0. *Can. J. Phys.* **91**, 444–449 (2013).

[18] Kojima, K., Katayama, N., Tamura, S., Shiomi, M. & Sawa, H. Vanadium trimers randomly aligned along the *c*-axis direction in layered LiVO_2_. *Phys. Rev. B* **100**, 235120 (2019).

[19] Kojima, K. et al. Short-range order and increased transition temperature in LiVO_2_ with weakened trimer frustration. *Phys. Rev. B* **107**, L020101 (2023).

[20] Kinemuchi, Y., Masuda, Y., Ozaki, K. & Fujita, A. LiVO_2_ as a new solid-state phase change material. *J. Alloy. Compd.* **882**, 160741 (2021).

[21] Pen, H. F., van den Brink, J., Khomskii, D. I. & Sawatzky, G. A. Orbital ordering in a two-dimensional triangular lattice. *Phys. Rev. Lett.* **78**, 1323–1326 (1997).

[22] Pen, H. F. et al. Phase transition in LiVO_2_ studied by near-edge x-ray-absorption spectroscopy. *Phys. Rev. B* **55**, 15500–15505 (1997).

[23] Ezhov, S. Y., Anisimov, V. I., Pen, H. F., Khomskii, D. I. & Sawatzky, G. A. Orbital polarization in LiVO_2_ and NaTiO_2_. *Europhys. Lett.* **44**, 491 (1998).

[24] Yoshitake, J. & Motome, Y. Trimer formation and metal-insulator transition in orbital degenerate systems on a triangular lattice. *J. Phys. Soc. Jpn.* **80**, 073711 (2011).

[25] Jin-no, T., Shimizu, Y., Itoh, M., Niitaka, S. & Takagi, H. Orbital reformation with vanadium trimerization in *d*^2^ triangular lattice LiVO_2_ revealed by ^51^V NMR. *Phys. Rev. B* **87**, 075135 (2013).

[26] Subedi, A. Mott-to-goodenough insulator-insulator transition in LiVO_2_. *Phys. Rev. B* **95**, 214119 (2017).

[27] Kikuchi, J. et al. ^51^V Knight shift and quadrupole interaction in the low-temperature phase of LiVO_2_. *J. Phys. Soc. Jpn.* **60**, 3620–3624 (1991).

[28] Thackeray, M., de Picciotto, L., David, W., Bruce, P. & Goodenough, J. Structural refinement of delithiated LiVO_2_ by neutron diffraction. *J. Solid State Chem.* **67**, 285–290 (1987).

[29] Carlson, O. N. & Owen, C. V. Preparation of high-purity vanadium metalb by the iodide refining process. *J. Electrochem. Soc.* **108**, 88 (1961).

