# Supplementary material for: Direct observation of V-trimers in the crystal structure of LiVO2
Source: Commun Chem. 2025 Jul 9;8:202. doi: 10.1038/s42004-025-01595-y (PMC12238393; doi:10.1038/s42004-025-01595-y)
Supplement: Supplementary file 1 — Supplementary Information [file 42004_2025_1595_MOESM1_ESM.pdf]

Suppl. Materials for "Direct observation of V-trimers in the crystal structure of $LiVO_2$"

S. Yun,[1] C.-F. Chang,[1] S.-H. Chen,[1,2] C.-Y. Kuo,[1,2,3] L. H. Tjeng,[1] and A. C. Komarek[1,*]

[1]*Max-Planck-Institute for Chemical Physics of Solids, Nöthnitzer Str. 40, D-01187 Dresden, Germany*
[2]*National Synchrotron Radiation Research Center, 101 Hsin-Ann Road, 30092 Hsinchu, Taiwan*
[3]*Department of Electrophysics, National Yang Ming Chiao Tung University, 30010 Hsinchu, Taiwan*

Crystal	$Li_{0.91}VO_2$
density	4.3926 g/cm^3
Temperature	room temperature
Wavelength	Mo K_α
Crystal system	trigonal
Space group	$P\,3_1$ (144)
Unit cell dimensions	a = 4.9240(5) Å
	b = 4.9240(5) Å
	c = 14.8130(15) Å
Volume	311.03(8) Å^3
Z	9
F(000)	385
Crystal size	~10-20 μm
$2\Theta_{max}$	70.8°
Index range	h: -7 → 8
	k: -7 → 7
	l: -23 → 19
Reflections in total / independant	14665 / 1564
Observed reflections / independant	11522 / 1445
R_σ (obs/all)	7.41% / 7.64%
R_{int} (obs/all)	6.69% / 6.72%
Redundancy	9.38
Completeness up to 2Θ = 68.1°	98%
Absorption correction	multi-scan
Min. / max. transmission	0.5216 / 0.7470
Refinement method	least squares on F^2
Goodness of fit	1.37
R / R_w ($I > 3\sigma(I)$)	4.80% / 12.83%
R / R_w (all)	6.05% / 14.06%

TABLE S1. Crystallographic & structural refinement data of a single crystal X-ray diffraction measurement of $Li_{0.91}VO_2$. The crystallographic software *Jana* was used for the structural refinement [1]. The twin fraction amounts to 49.972(23)%.

**atom**	**occ.**	**x**	**y**	**z**	U_{iso} (Å^2)
Li1	0.950(6)	0.5455(16)	0.4440(14)	0.3611(6)	0.0210(7)
Li2	0.944(5)	0.1883(19)	0.0976(13)	0.0254(4)	0.0210(7)
Li3	1	0.1812(14)	0.7570(14)	0.3607(5)	0.0210(7)
V1	1	0.24607(14)	0.48700(11)	0.19373(17)	0.00451(6)
V2	1	0.54468(11)	1.0765(2)	0.19557(17)	0.00451(6)
V3	0.760(3)	0.8546(3)	0.7649(4)	0.19266(17)	0.00451(6)
V4	0.240(3)	0.9577(7)	0.7980(5)	0.1997(2)	0.00451(6)
V5	0.050(6)	0.5455(16)	0.4440(14)	0.3611(6)	0.0210(7)
V6	0.056(5)	0.1883(19)	0.0976(13)	0.0254(4)	0.0210(7)
O1	1	0.5535(6)	0.7725(7)	0.2806(2)	0.00573(13)
O2	1	0.2224(10)	1.1144(5)	0.2670(2)	0.00573(13)
O3	1	0.8797(6)	0.4356(7)	0.2709(3)	0.00573(13)
O4	1	0.5513(11)	0.4389(5)	0.1202(3)	0.00573(13)
O5	1	0.2127(7)	0.7841(10)	0.1159(2)	0.00573(13)
O6	1	0.2221(15)	0.1087(7)	0.7882(3)	0.00573(13)

TABLE S2. Refinement results of single crystal X-ray diffraction measurements of $Li_{0.91}VO_2$. A split-atom model was used (V3 and V3'). Vx1 and Vx2 are additional vanadium ions within the Li-deficient layers; there was no indication for a Li deficiency at the Li3 site. The Li:V ratio amounts to 0.932(4) which is close to the value expected from the c/a-ratio of $Li_{0.91}VO_2$ (following the literature values of the lattice constants in Ref. [2] that was determined by inductively coupled plasma optical emission spectrometry).

* Komarek@cpfs.mpg.de

Crystal	$Li_{1.0}VO_2$
density	4.3294 g/cm^3
Temperature	room temperature
Wavelength	Mo K_α
Crystal system	trigonal
Space group	$P\,3_1$ (144)
Unit cell dimensions	a = 4.9096(3) Å
	b = 4.9096(3) Å
	c = 29.7092(24) Å
Volume	620.186(126) Å^3
Z	18
F(000)	756
Crystal size	~10-20 μm
$2\Theta_{max}$	56.5°
Index range	h: -6 → 6
	k: -6 → 6
	l: -39 → 39
Reflections in total / independant	10458 / 2024
Observed reflections / independant	8393 / 1746
R_σ (obs/all)	5.73% / 5.95%
R_{int} (obs/all)	4.44% / 4.53%
Redundancy	5.17
Completeness up to $2\Theta_{max}$	99.62%
Absorption correction	multi-scan
Min. / max. transmission	0.4589 / 0.7457
Refinement method	least squares on F^2
Goodness of fit	2.03
R / R_w ($I > 3\sigma(I)$)	5.63% / 16.72%
R / R_w (all)	7.00% / 17.34%

TABLE S3. Crystallographic & structural refinement data of a single crystal X-ray diffraction measurement of $LiVO_2$. The crystallographic software *Jana* was used for the structural refinement [1]. The twin fraction amounts to 49.53(14)%.

**atom**	**occ.**	**x**	**y**	**z**	U_{iso} (Å^2)
V1	1	0.5884(6)	0.4840(4)	0.66125(8)	0.0010(1)
V2	1	0.1899(4)	0.7717(4)	0.66102(6)	0.0010(1)
V3	0.719(8)	0.8957(5)	1.0703(8)	0.66107(10)	0.0010(1)
V3'	0.281(8)	0.2464(16)	0.0692(12)	0.32814(14)	0.0010(1)
V4	1	0.5166(4)	0.7519(4)	0.82755(7)	0.0010(1)
V5	1	0.9266(4)	1.4485(4)	0.82746(6)	0.0010(1)
V6	1(0)	0.2165(6)	0.1487(7)	0.82772(7)	0.0010(1)
O1	1	0.8757(17)	0.785(2)	0.8617(2)	0.0049(3)
O2	1	0.546(2)	0.1075(16)	0.8624(3)	0.0049(3)
O3	1	0.5558(19)	0.7957(19)	0.7001(3)	0.0049(3)
O4	1	0.9009(18)	0.444(3)	0.6992(3)	0.0049(3)
O5	1	0.225(2)	0.4489(18)	0.8731(2)	0.0049(3)
O6	1	1.221(3)	1.120(3)	0.6995(3)	0.0049(3)
O7	1	0.210(2)	0.7645(19)	0.2896(2)	0.0049(3)
O8	1	0.2063(18)	0.4229(16)	0.1214(3)	0.0049(3)
O9	1	0.567(2)	0.121(2)	0.1210(3)	0.0049(3)
O10	1	0.238(3)	0.115(3)	0.4545(3)	0.0049(3)
O11	1	0.567(2)	0.437(3)	0.2919(3)	0.0049(3)
O12	1	0.218(2)	0.1024(17)	0.9504(3)	0.0049(3)
Li1	1	0.547(4)	0.437(5)	0.4048(6)	0.0201(17)
Li2	1	0.224(7)	0.111(5)	0.0781(4)	0.0201(17)
Li3	1	0.246(5)	0.776(4)	0.4025(5)	0.0201(17)
Li4	1	0.524(4)	0.118(6)	0.2476(8)	0.0201(17)
Li5	1	0.167(4)	0.742(4)	0.9129(6)	0.0201(17)
Li6	1	0.230(7)	0.110(5)	0.5785(6)	0.0201(17)

TABLE S4. Refinement results of single crystal X-ray diffraction measurements of $LiVO_2$. A split-atom model was used (V3 and V3'). There was no clear indication for a split site in every other vanadium layer.

space group	Laue symmetry	$LiVO_2$ R_{int} (obs/all)	$Li_{0.91}VO_2$ R_{int} (obs/all)
P1	$\bar{1}$	3.52%/3.60%	5.73%/5.79%
$P\bar{1}$	$\bar{1}$	3.89%/3.97%	6.38%/6.42%
$P\bar{3}$	$\bar{3}$	4.68%/4.75%	7.06%/7.07%
P3	$\bar{3}$	4.45%/4.54%	6.69%/6.72%
**$P3_2$**	$\bar{3}$	**4.44%/4.53%**	**6.69%/6.72%**
**$P3_1$**	$\bar{3}$	**4.44%/4.53%**	**6.69%/6.72%**
$P\bar{3}1c$	$\bar{3}1m$	4.80%/4.86%	–
P31c	$\bar{3}1m$	4.62%/4.69%	–
$P\bar{3}1m$	$\bar{3}1m$	4.85%/4.92%	7.14%/7.15%
P31m	$\bar{3}1m$	4.66%/4.74%	6.81%/6.82%
P312	$\bar{3}1m$	4.77%/4.84%	7.09%/7.10%
P3212	$\bar{3}1m$	4.76%/4.84%	7.09%/7.10%
P3112	$\bar{3}1m$	4.76%/4.84%	7.09%/7.10%

TABLE S5. List of space groups for $Li_{1-x}VO_2$ that could be considered regarding their R_{int}-values. Space groups of other Laue symmetries that are not listed here (like *P3m1* etc.) would result in R_{int}-values > 50%. The space groups that have smallest R_{int}-values (for trigonal symmetries) and that lead to the best GoF and R-values within the structure refinement are highlighted in bold.

Supplementary References

[1] V. Petříček, M. Dušek and L. Palatinus, Crystallographic Computing System JANA2006: General features, Zeitschrift für Kristallographie - Crystalline Materials **229** 345–352 (2014)

[2] Y. Kinemuchi, Y. Masuda, K. Ozaki and A. Fujita, $LiVO_2$ as a new solid-state phase change material, Journal of Alloys and Compounds **882**, 160741 (2021)

Appendix A: Checkcif

Checkcif was run with validation type 'Validation of CIF only (no structure factors)' to identify 'Level A and B' alerts in the CIF files.

1. $Li_{0.91}VO_2$

The A and B level alerts from Checkcif, along with our comments (author response), are listed below for the $Li_{0.91}VO_2$ sample.

The following ALERTS were generated. Each ALERT has the format
test-name_ALERT_alert-type_alert-level .
Click on the hyperlinks for more details of the test.

Alert level A
ATOM007_ALERT_1_A _atom_site_aniso_label is missing
Unique label identifying the atom site.

Author Response: Li is a too light element to be refined in anisotropic approximation, and also V ions should be treated with Uiso since a split atom model is used, and finally also O is a lighter element and there is a large amount of oxygen ions (sites) present in the unit cell, (plus the effect of twinning etc.).

PLAT097_ALERT_2_A Large Reported Max. (Positive) Residual Density 10.55 eA-3

Author Response: Reflections are distinctly boradened in c*-direction - thus, disorder is present in the real crystal. With this disorder one can not expect ideally perfect values as would be expected for a perfect long range ordered crystal without disorder.

Alert level B
DIFMN02_ALERT_2_B The minimum difference density is < -0.1□ZMAX□1.00
_refine_diff_density_min given = -3.150
Test value = -2.300

Author Response: Reflections are distinctly boradened in c*-direction - thus, disorder is present in the real crystal. With this disorder one can not expect ideally perfect values as would be expected for a perfect long range ordered crystal without disorder.

PLAT021_ALERT_4_B Ratio Unique / Expected Reflections too High ... 15.871
Unique Refl = 14665 / # Expected Refl = 924

Author Response: The data were not averaged, the refined data set was collected using a twinned specimen.

PLAT098_ALERT_2_B Large Reported Min. (Negative) Residual Density -3.15 eA-3
V1 V2 V3 O1 O2 O3 O4 O5
O6

Author Response: Reflections are distinctly boradened in c*-direction - thus, disorder is present in the real crystal. With this disorder one can not expect ideally perfect values as would be expected for a perfect long range ordered crystal without disorder.

2. $LiVO_2$

The A and B level alerts identified by Checkcif, along with our corresponding comments (author response), are presented below for the $LiVO_2$ sample.

The following ALERTS were generated. Each ALERT has the format
test-name_ALERT_alert-type_alert-level .
Click on the hyperlinks for more details of the test.

Alert level A
ATOM007_ALERT_1_A _atom_site_aniso_label is missing
Unique label identifying the atom site.

Author Response: Li is a too light element to be refined in anisotropic approximation, and also V ions should be treated with Uiso since a split atom model is used, and finally also O is a lighter element and there is a large amount of oxygen ions (sites) present in the unit cell, (plus the effect of twinning etc.).

PLAT097_ALERT_2_A Large Reported Max. (Positive) Residual Density 7.38 eA-3
V1 V2 V3 O3 O4 O6 O7 O11
O12 V4 V5 V6 O1 O2 O5 O8
O9 O10

Author Response: Reflections are distinctly broadened in c*-direction - thus, disorder is present in the real crystal. With this disorder one can not expect ideally perfect values as would be expected for a perfect long range ordered crystal without disorder.

Alert level B
DIFMN02_ALERT_2_B The minimum difference density is < -0.1□ZMAX□1.00
_refine_diff_density_min given = -2.400
Test value = -2.300

Author Response: Reflections are distinctly broadened in c*-direction - thus, disorder is present in the real crystal. With this disorder one can not expect ideally perfect values as would be expected for a perfect long range ordered crystal without disorder.

PLAT021_ALERT_4_B Ratio Unique / Expected Reflections too High ... 10.027
Unique Refl = 10458 / # Expected Refl = 1043

Author Response: The data were not averaged, the refined data set was collected using a twinned specimen.

PLAT098_ALERT_2_B Large Reported Min. (Negative) Residual Density -2.40 eA-3

Author Response: Reflections are distinctly broadened in c*-direction - thus, disorder is present in the real crystal. With this disorder one can not expect ideally perfect values as would be expected for a perfect long range ordered crystal without disorder.
