# Supplementary material for: Direct observation of V-trimers in the crystal structure of LiVO2
Source: Commun Chem. 2025 Jul 9;8:202. doi: 10.1038/s42004-025-01595-y (PMC12238393; doi:10.1038/s42004-025-01595-y)
Supplement: Supplementary file 2 — Description of Additional Supplementary Files [file 42004_2025_1595_MOESM2_ESM.pdf]

Description of Additional Supplementary Files

File name- Supplementary Data 1

File description- CIF file of $Li_{0.91}VO_2$

File name- Supplementary Data 2

File description- CIF file of $LiVO_2$

File name- Supplementary Data 3

File description- The numerical data of the graphs/plots
